# Supplementary material for: Conditional chemoconnectomics (cCCTomics) as a strategy for efficient and conditional targeting of chemical transmission
Source: eLife. 2024 Apr 30;12:RP91927. doi: 10.7554/eLife.91927 (PMC11060718; doi:10.7554/eLife.91927)
Supplement: Figure 3—source data 3. [file elife-91927-fig3-data3.zip › Figure 3 source data3 part 1.zip!/Figure 3-Source data 3-part1/1063-13-CRISPResso_Report_8cz_fsdr.zip!/CRISPResso_on_1063_15/1c.Alignment_barplot.pdf]

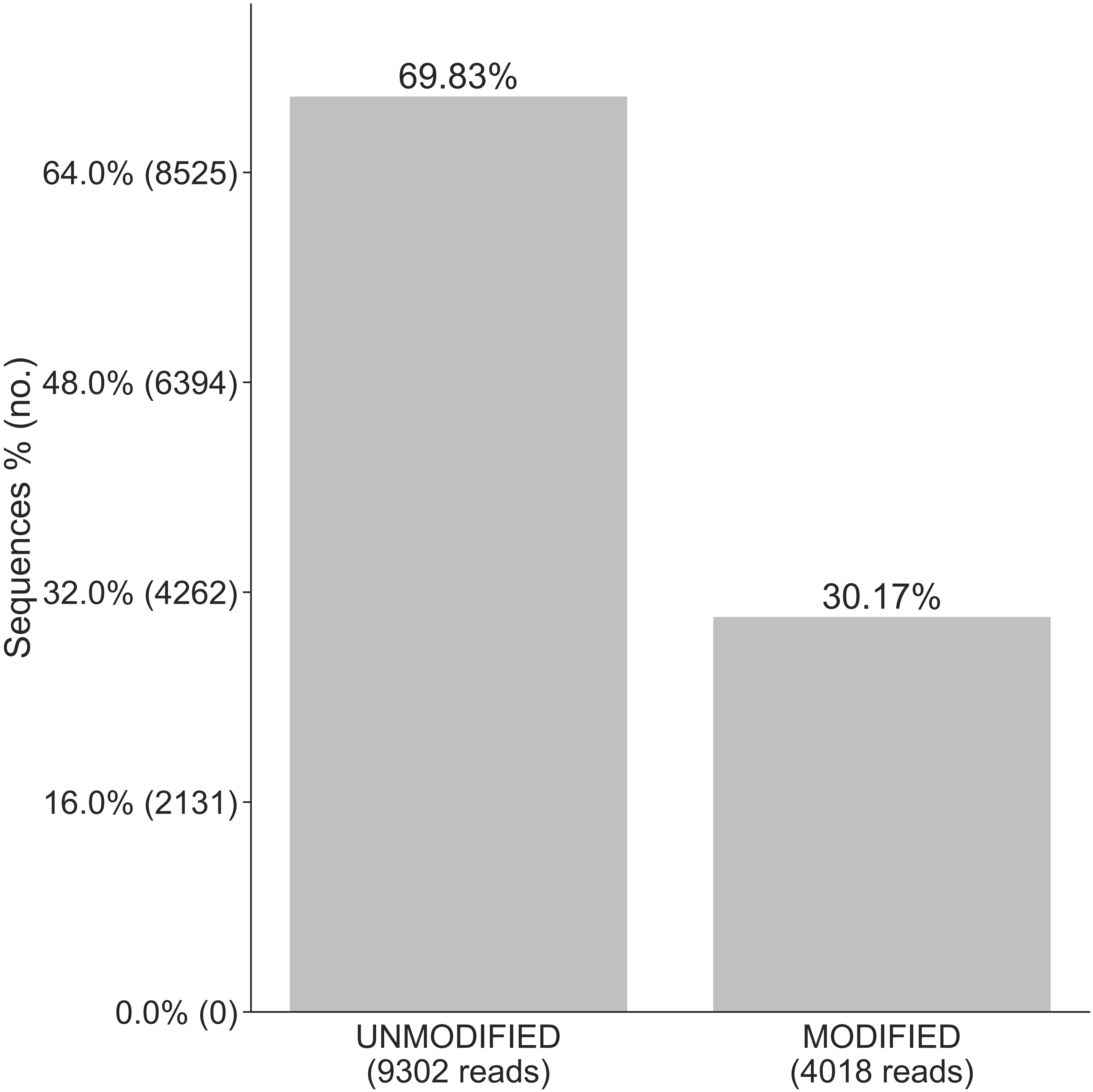
69.83%
30.17%
Sequences % (no.)
64.0% (8525)
48.0% (6394)
32.0% (4262)
16.0% (2131)
0.0% (0)
UNMODIFIED
(9302 reads)
MODIFIED
(4018 reads)
